# Identification of Development-Related Genes in the Ovaries of Adult *Harmonia axyridis* (Pallas) Lady Beetles Using a Time- Series Analysis by RNA-seq

**DOI:** 10.1038/srep39109

**Published:** 2016-12-14

**Authors:** Wenxiao Du, Fanrong Zeng

**Affiliations:** 1 Institute of Plant Protection, Chinese Academy of Agricultural Sciences, Key Laboratory of Integrated Pest Management in Crops, Ministry of Agriculture, Beijing 100081, PR China

## Abstract

Adults of the lady beetle species *Harmonia axyridis* (Pallas) are bred artificially en masse for classic biological control, which requires egg-laying by the *H. axyridis* ovary. Development-related genes may impact the growth of the *H. axyridis* adult ovary but have not been reported. Here, we used integrative time-series RNA-seq analysis of the ovary in *H. axyridis* adults to detect development-related genes. A total of 28,558 unigenes were functionally annotated using seven types of databases to obtain an annotated unigene database for ovaries in *H. axyridis* adults. We also analysed differentially expressed genes (DEGs) between samples. Based on a combination of the results of this bioinformatics analysis with literature reports and gene expression level changes in four different stages, we focused on the development of oocyte reproductive stem cell and yolk formation process and identified 26 genes with high similarity to development-related genes. 20 DEGs were randomly chosen for quantitative real-time PCR (qRT-PCR) to validate the accuracy of the RNA-seq results. This study establishes a robust pipeline for the discovery of key genes using high-throughput sequencing and the identification of a class of development-related genes for characterization.

The multi-coloured Asian lady beetle *Harmonia axyridis* (Pallas) is a well-known and highly efficient predator of aphids, and other insect pests of fruit trees, flowers, and vegetables^1^,^2^,^3^. Extensive research has focused on aspects of *H. axyridis*, such as genetics or evolutionary responses to population dynamics^4^, biological control^4^, life history^5^, biology^6^, and potential non-target impacts^4^. As an important part of previous research, *H. axyridis* has been extensively released for classic biological control worldwide^4^ (e.g., eastern Asia, Europe, Africa, and South and North America) with many significant benefits^4^,^7^. The ovary is an import reproductive organ in *H. axyridis* and typically associated with the egg-laying ability of *H. axyridis* adults. Therefore, the ovary contributes to the prosperity of the population and influences mass artificial breeding for classic biological control^8^. Ovary development can be divided into the following four development stages according to the presence of egg rooms and egg yolk deposition: the undifferentiated stage (Stage 1), ovarian developmental growth (Stage 2), the yolk sedimentary period (Stage 3) and early maturity for maternity (Stage 4)^8^. Previous studies have been performed at the phenotypic level, and few studies focused on understanding the key biological processes based on the *H. axyridis* transcriptome, especially among the ovary developmental genes. To understand the molecular mechanisms underlying ovary development, improve the artificial reproductive capacity of *H. axyridis* from the perspective of genes for biological control, and lay a foundation for other basic studies, it is indispensable to detect development-related genes in the ovarie of *H. axyridis* adult.

RNA-seq is a convenient and accurate technology that has been adopted to study gene function in many eukaryotes^9^. Following its rapid development, RNA-seq has been used to detect the genome sequences of many species rapidly with relatively high accuracy, extension and cost performance, resulting in great breakthroughs in biological research^10^. The research applications include basic research, clinical diagnosis and drug development^11^. RNA-seq provides a far more precise measurement of transcript levels and their isoforms than other methods^12^. Vera (2008) first reported the *Melitaea cinxia*. L transcriptome sequence obtained with RNA-seq, which was considered as paradigm in insects based on the new generation of transcriptome sequencing technology^13^. Gibbons (2009) sequenced *Anopheles gambiae.* L and *Aedes aegypti.* L, which confirmed that the short-read sequencing platform was feasible in non-model insect transcriptome studies^14^. Malone and Oliver (2011) adopted Illumina to perform RNA-seq experiments with *Drosophila pseudoobscura*. L and confirmed that this sequencing technology was reliable for gene expression research^15^.

In our study, we applied RNA-seq to obtain ovary transcriptomes from *H. axyridis* adults in Stages (1–4) (S1–S4), and performed an in depth analysis of these data to detect development-related genes. Additionally, we performed quantitative reverse transcription (qRT-PCR) analysis to validate the RNA-seq results. This transcriptome-wide study of mRNAs in the *H. axyridis* ovary will facilitate the study of the functions of development-related genes and provide potential RNAi targets to accelerate the breeding of *H. axyridis* for use in biological control.

## Results

### Transcriptome sequencing and read assembly

After Illumina Solexa deep sequencing, 21.5 Gb of clean data were obtained from the transcriptome sequencing of the *H. axyridis* adult ovary. To obtain an overview of the ovary expression profile at the different developmental stages in *H. axyridis* adults, four expressed gene libraries (S1: 22,364, 281; S2: 18, 460,991; S 3: 20,636,344; and S4: 24,531,820) containing clean reads were obtained for analysis. All base calling rates were greater than 92% (Table 1). A large number of reads was distributed at approximately 500 bp, with lengths ranging from 300 to 2000 bp. After assembly, 64,231 transcripts and 47,550 unigenes were obtained; the N50s of the transcripts and unigenes were 1448 and 1282 bp, respectively (Table 2), indicating high integrity assembly.

### Unigene annotation

For annotation of the unigenes, BLAST software (version 2.2.26)^16^ was used to compare the unigene sequences with the Clusters of Orthologous Groups (COG)^17^, Gene Ontology (GO)^18^, Kyoto Encyclopedia of Genes and Genomes (KEGG)^19^, euKaryotic Orthologous Groups (KOG)^20^, Protein family (Pfam)^21^, Swiss-Prot^22^, and non-redundant (nr) databases^23^ to annotate the unigenes. Functions were predicted and classified with the COG classification system, resulting in the annotation of 7296 unigenes (Table 3). Additionally, 12,985 unigenes were annotated by the GO classification system. The pathway involvement of 8144 unigenes identified in this study was predicted based on a comparison with the KEGG database. A total of 17,235, 16,822, 16,863, and 28,186 unigenes were analysed with the KOG, Pfam, Swiss-Prot, and nr databases, respectively. Overall, 28,558 unigenes were annotated by selecting parameters with an E value ≤1e-5 (Table 3), to obtain an unigene annotation database for ovaries in *H. axyridis* adults (Supplementary Dataset File 1).

### Differentially expressed genes (DEGs) analysis

To detect specific target genes that affected the developmental process, we performed a series of genome-wide expression profiling comparisons to examine gene activity changes between S1 and the other three stages. An Fragments per kilobase of exon model per million mapped reads (FPKM) fold change >2 and a false discovery rate (FDR) <0.05 were used as the thresholds to identify significant differences in gene expression.

**Figure 1:**
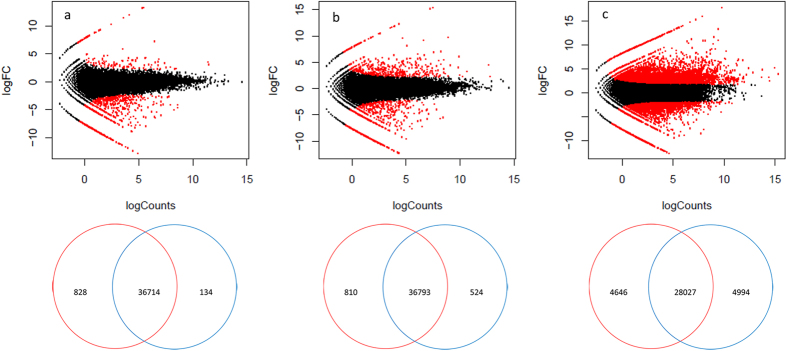
Scatter diagrams representing genome-wide expression profiling comparisons between S1 and the other three stage. Note: Each black point represents a non-significant difference in gene expression, and each red point indicates a significant differentially expressed gene. Abscissa: log10 (Counts); Ordinate: log2 (FC) expression difference multiple values between two gene samples. a: S1vs. S2, b: S1vs. S3, c:S1vs. S4.

Comparing S2 with S1, 962 genes were differentially expressed, including 134 up-regulated genes and 828 down-regulated genes (Table 4). In S3 compared with S1, the expression of 524 genes was up-regulated and the expression of 810 genes was down-regulated (Table 4). When the gene expression levels were compared between S1 and S4, 4994 genes were up-regulated and 4646 genes were down-regulated, for a total number of 9640 genes (Table 4). These results revealed that the number of DEGs increased as development proceeded (Fig. 1). All of the obtained DEGs were combined into one DEG database (Supplementary Dataset File 2).

### Identification and sequence analysis of development-related genes in a time series from the *H. axyridis* adult ovary

**Figure 2:**
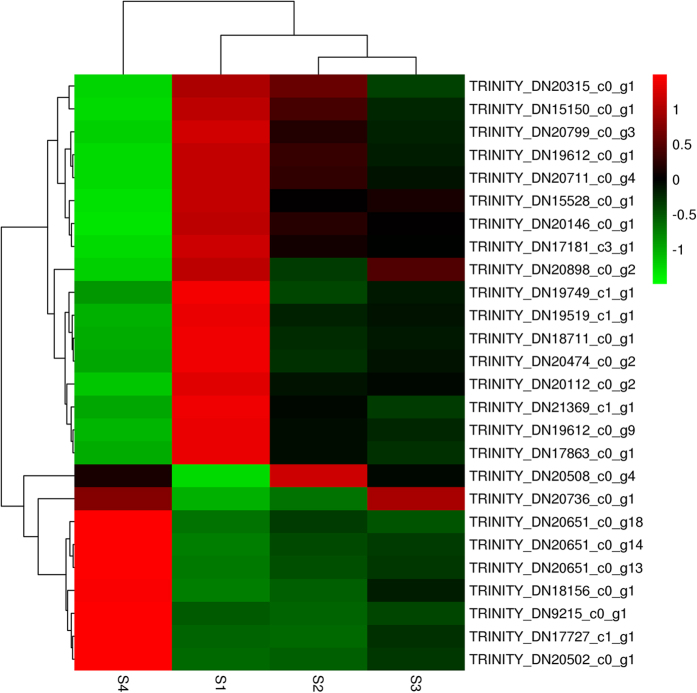
Clustering of development-related genes. The colour represents the gene expression levels in the samples in different columns. Different columns represent different samples, and different lines represent different genes.

We combined the unigene annotations from the unigene annotation databases obtained above (Supplementary Dataset File 1), literature reports^24^,^25^,^26^,^27^,^28^,^29^,^30^,^31^, and transcription level changes in the four different stages (i.e., preference was given to genes that were up- or down- regulated continuously from S1 to S4) to identify genes associated with the formation of the polar plasm, the signal transduction mechanism in the ovary microenvironment and yolk formation, which define the development of the insect ovary^31^. In total, we identified 26 unigenes with high similarity to development-related genes (Table 5). All of them can be annotated in nr and divided into the following eight groups based on specific molecular functions: one gene related to polar formation, two genes related to bone morphogenetic protein function related, one gene related to DE-cadherin function related, two genes related to insulin related function, one gene related to ecdysone signalling pathways, 11 genes related to Notch signalling pathway, five genes related to Jak-STAT signalling pathway, and threegenes related to vitellogenin function. One gene related to polar formation was predicted to be full-length and to contribute to embryonic polarity protein dorsal-like isoform X1. Two putative sequences associated with bone morphogenetic protein functions were also predicted to be full-length. Their nucleotide lengths ranged from 3, 186 bp to 4, 758 bp, which corresponded to protein lengths of 517 aa to 821 aa, and the genes contained annotations for the bone morphogenetic protein receptor. TRINITY_DN17181_c3_g1 was the only gene related to DE-cadherin functions after screening and was annotated as a DE cadherin-like protein. Two genes related to insulin function were identified; neither gene was full length, and the genes contained annotations for the insulin receptor substrate. One gene was annotated as an ecdysone-induced protein included in the ecdysone signalling pathway. Of the 11genes predicted to be related to the Notch signalling pathway, six genes were full-length. All homologous functions from the nr database are shown in Table 5. In this study, we identified five genes related to the Jak-STAT signalling pathway; four of these genes were full-length, and all of the genes were predicted to possess different homologous functions. Three genes were related to vitellogenin functions, which plays an important role in yolk formation. TRINITY_DN20508_c0_g4 was annotated as the vitellogenin receptor. TRINITY_DN20502_c0_g1 and TRINITY_DN20736_c0_g1 were annotated as vitellogenin. In total, 18 unigenes in the *H. axyridis* adult ovary could be blasted against *Tribolium castaneum* Herbst, which accounted for 69.2% of all identified unigenes with high similarity to development-related genes. The others genes blasted to *Anoplophora glabripennis* Motschulsky (15.4%), *Agrilus planipennis*Fairmaire (11.5%) and *Polistes canadensis*L (3.8%) (Table 5). Heat maps of the development-related genes identified in the ovaries of *H. axyridis* adults during development indicated that the same gene had different expression levels in different stages (Fig. 2).

### Functional annotation clustering of development-related genes

**Figure 3:**
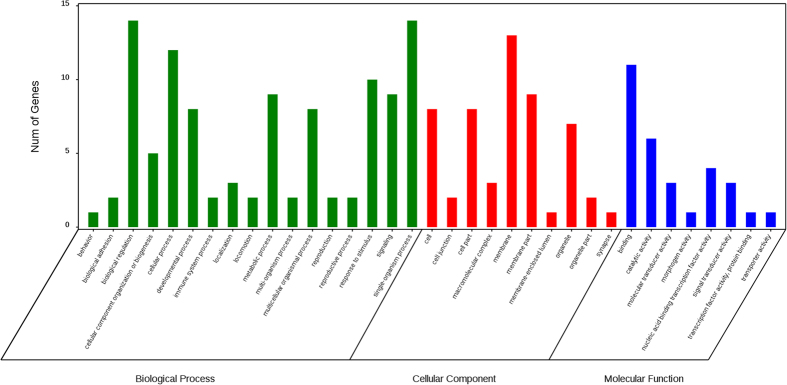
GO annotation classification of development-related genes converted to unigenes annotation database of ovaries in *H. axyridis* adult. The abscissa is the GO classification, the ordinate left is the gene number percentage, and the right is the number of genes.

**Figure 4:**
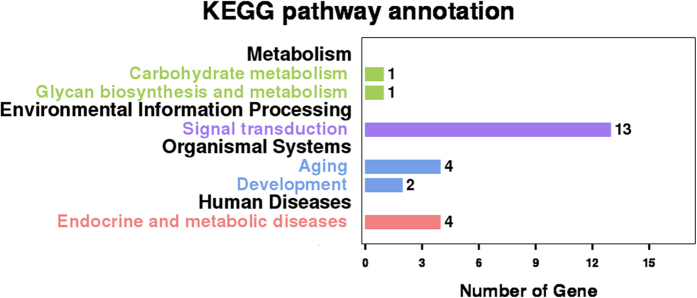
KEGG pathway annotation classification of development-related genes converted to unigenes annotation database of ovaries in *H. axyridis* adult. The abscissa is the gene number, and the ordinate left is the KEGG classification.

Following GO annotation, the 26 development-related gene were classified into 35 different groups belonging to three main categories (“biological process”, “cellular component”, and “molecular function”) (Fig. 3). “Biological regulation” (14 genes), “single-organism process” (14 genes), and “cellular process” (12 genes) were the top three abundant categories in “biological process”, whereas “membrane” (13 genes),“membrane part” (9 genes), “cell” (8 genes), “cell part” (8 genes) and “organelle” (7 genes) were the top five categories in “cellular component”. Gene number involved in “binding” (11 genes) was the dominant in the “molecular function” category (Fig. 3) (Supplementary information Table 1). A total of 21 development-related genes were annotated by the KEGG classification system, including 13 genes related to signal transduction, four genes for ageing or endocrine and metabolic diseases, two genes for development, and one gene for carbohydrate metabolism or glycan biosynthesis and metabolism (Fig. 4) (Supplementary information Table 2).

### qRT-PCR validation of the DEGs results

**Figure 5:**
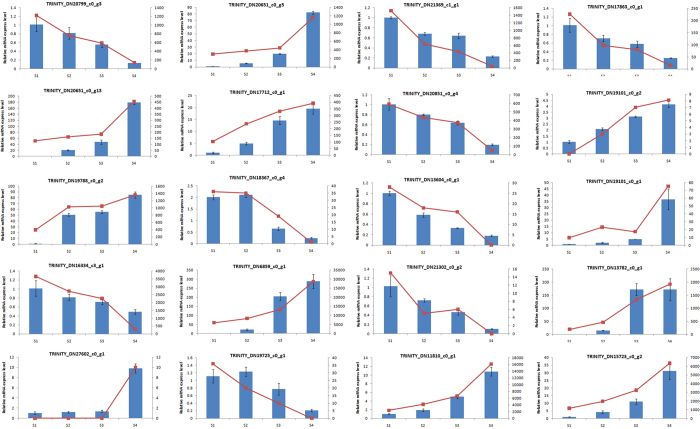
The correlation between mRNA expression levels using qPCR and mRNA sequencing. The blue bar denotes expression values based on qPCR, and the red line denotes expression values based on mRNA sequencing.

To validate our DEG results, 20 randomly selected genes were analysed by qRT-PCR. Primer pairs for qRT-PCR were designed based on the nucleotide sequences of the transcriptome results. β-Actin was chosen as the internal control. The expression changes of these genes in S1, S2, S3 and S4 ovaries are shown in Fig. 5. Expression patterns similar to the DEG results were obtained. In combination with the previous study, the accuracy of the DEG results was verified^8^ (Fig. 5).

## Discussion

The ovary is a reproductive response organ that plays a crucial role in population breeding and undergoes dynamic and molecular changes. The development mechanism of the ovary has been studied in some insects. Liu (2015) studied the function of the vitellogenin gene in *Chrysopa septempunctata.* Wesmael^32^. Based on the sequence alignment with the results in this study, the TRINITY_DN20736_c0_g1 sequence shows 46.52% similarity with the vitellogenin gene reported in the study of Liu (2015)^32^. This finding suggests that these two genes have a certain similarity. Xie (2015) screened two genes related to polar cell formation; however, their sequence similarity was 6.96% and 14.82% with TRINITY_DN20651_c0_g14 identified in this study, indicating that these genes are not similar^31^. Based on the RNA-seq results, approximately 405 cellular-related genes were identified during ovary development in *Bombyx mori.* L in the fifth instar and early pupal stages, thus providing new insights into our understanding of the molecular mechanism of ovary development and oogenesis in the silkworm^33^. However, ovarian development related to natural enemy insects has mostly remained at the anatomic level^11^. *H. axyridis* is an important natural enemy. The ovary of *H. axyridis* has obvious spatial and temporal characteristics, and all four developmental stages are regulated by developmental gene expression^4^. Consequently, a comprehensive understanding of the molecular mechanisms underlying the regulation of ovary growth and development is very important for improving the reproductive capacity of *H. axyridis*.

Previous reports suggest that the formation and differentiation of germline stem cells (GSCs) directly determine the insect ovipositor^34^. GSCs develop from primordial germ cells (PGCs), which also maintain the balance of differentiation in the microenvironment^35^. In this series of physiological activities, the formation of polar plasm decides the development of the PGCs^31^. In this study, TRINITY_DN20651_c0_g14 was annotated as embryonic polarity protein dorsal-like isoform X1 and might have participated in the formation of polar cells in the ovaries, similar to XP_015172818.1 in *Polistes canadensis*L (Table 5). This process might represent the basic conditions of cellular differentiation^36^ and organismal zygote development^37^. The signal transduction mechanism maintains the stability of the microenvironment^38^. Six groups of genes related to signal transduction were identified. Among them, bone morphogenetic protein function-related genes are dominant in maintaining GSC quality^31^. Two of these genes were annotated for the bone morphogenetic protein receptor, similar to the report of Yamashita^39^ (Table 5). TRINITY_DN17181_c3_g1 was predicted to be a DE cadherin-like proteinrelated to GSC activity^29^. TRINITY_DN19612_c0_g1 and TRINITY_DN19612_c0_g9 are related to the insulin receptor, which affects insulin signalling pathways and controls the microenvironment via upstream regulation, thereby affecting GSC development^31^. TRINITY_DN20112_c0_g2 was the only gene related to the ecdysone signalling pathways and plays an important role in maintaining the stability of the microenvironment and GSC differentiation^31^. In the organization of many species, insulin signalling pathways and ecdysone signalling pathways correlate with one another^40^. The 11 genes related to the Notch signalling pathway may regulate the cell cap. Deficiency of the Notch signalling pathway might lead to a decrease in GSCs^26^. Five genes were included in the Jak-STAT signalling pathway, which can affect the bone morphogenetic protein^25^. Three genes were related to vitellogenin, which provides nutrition to the ovary^31^ and thus is indispensable for embryonic development^41^.

TRINITY_DN20502_c0_g1 and TRINITY_DN20736_c0_g1 were predicted to be vitellogenin, but the similarity between these genes was only 38.73%, indicating that they are different genes. The functional genes identified in this study are closely associated with ovarian development. The 18 (69.2%) development-related genes identified in this study could be blasted against *Tribolium castaneum* Herbst, indicating high homology between them, and four (15.4%), three (11.5%), and one (3.8%) development-related genes could be blasted against *Anoplophora glabripennis.* Motschulsky, *Agrilus planipennis.* Fairmaire and *Polistes Canadensis.* L, respectively, revealing evolutionary relationships among these species. Screening for functional genes could provide a foundation for research on developmental mechanisms in the *H. axyridis* adult ovary.

This study adopted functional transcriptome analysis with deep sequencing and the identification of genes after focusing on key ovary development processes, providing a new method to identify genes related to ovary development. Egg production might be stimulated in *H. axyridis* adults by interfering with development-related genes using the information obtained in this study. In a future study, a series of functional validation experiments will be performed to evaluate the development-related genes identified in this study.

## Materials and Methods

### Experimental insects

The *H. axyridis* used in this study were collected from the suburbs of Beijing and reared in the laboratory at 26±1 °C and 60–70% RH under a 16L:8D photoperiod for the experiments^42^. The insects were fed Acyrthosiphon pisum Harris reared on *Pisum sativum.* L. (Millborn Seeds, Zhewan 1, Beijing, China) in the laboratory. Ovary samples were collected from 100 *H. axyridis* adults on each of four typical dates, including the first day after emergence (Stage 1), the third day after emergence (Stage 2), the fourth day after emergence (Stage 3), and the seventh day after emergence (Stage 4)^8^.

### Total RNA preparation

Total RNA was extracted from each stage on the first, third, fourth, and seventh days after insect emergence using TRIzol (Reagent, Thermo Fisher Scientific, Waltham, USA) according to the manufacturer’s instructions^43^. Each RNA sample was incubated with RNase-free DNase I (Reagent, Thermo Fisher Scientific, Waltham, USA) for 30 minutes at 37 °C^43^. The total amount of RNA in each sample was approximately 30 μg. The RNA samples were isolated and purified from the RNA pool with an Oligotex mRNA Midi kit (Reagent, Qiagen, Dusseldorf, Germany). A NanoDrop ND-1000 spectrophotometer (Specialized equipment, Thermo Fisher Scientific, Waltham, USA) was used to measure the absorbance at 260/280 nm (A260/A280) to determine the quality and quantity of the purified RNA. The integrity of the RNA was verified by electrophoresis on a 1.5% (w/v) agarose gel.

### cDNA library construction and Illumina Solexa sequencing

A supported oligo ligation detection (SOLiD) Whole Transcriptome Analysis kit (Reagent, Thermo Fisher Scientific, Waltham, USA) was used to build a random fragment sequencing library following the manufacturer’s standard procedure^44^. The purified mRNA was fragmented with an interrupt reagent in a thermomixer and used as the template to synthesize first-strand cDNA. RNase H (Reagent, Thermo Fisher Scientific, Waltham, USA), DNA polymerase I (Reagent, Promega, Madison, USA), and dNTPs (Reagent, Thermo Fisher Scientific, Waltham, USA) were used to synthesize the second-strand cDNA. For ligation to sequence adapters, the cDNA was purified and repaired, and A bases were added to the 3′-end. Fragments of the appropriate size were selected and amplified by polymerase chain reaction (PCR) to construct the final sequencing library. After testing the quality control using an ABI Step One Plus Real-Time PCR System (Specialized equipment, Applied Biosystems, Foster, USA) and an Agilent 2100 Bioanalyzer (Specialized equipment, Agilent, Palo Alto, USA), the library was sequenced using the Illumina HiSeq 4000 platform (Specialized equipment, Illumina, SanDiego, USA)^44^.

### Quality control of sequencing data

The raw Solexa sequencing data were processed using in-house Perl scripts to remove low-quality reads, reads containing poly-Ns and reads containing adapters to obtain clean data^44^. Concurrently, the Q20, Q30, and GC contents were calculated for the clean data. Clean data of high quality were used for the following analyses^44^.

### De novo transcriptome assembly and quantification of gene expression levels

The trimmed reads were de novo assembled using trinityrnaseq_r20140717 with the default setting^45^. Bowtie v2.23 was used to map the clean reads to the Trinity assembly^46^. Then, RSEM^47^ was used to estimate the gene and isoform expression levels by calculating the FPKM of each gene and isoform (which considers the effect of the sequencing depth and gene length of the reads counted simultaneously)^48^.

### Unigene annotation

The gene functions were annotated using the COG (http://www.ncbi.nlm.nih.gov/COG/)^17^, GO (http://www.geneontology.org/)^18^, KEGG (http://www.genome.jp/kegg/)^19^, KOG (http://www.ncbi.nlm.nih.gov/KOG/)^20^, Pfam (http://pfam.xfam.org/)^21^, Swiss-Prot (http://www.uniprot.org/)^22^, and nr databases (ftp://ftp.ncbi.nih.gov/blast/db/)^23^. The annotations of the unigenes were com-bined to create a database of annotated unigenes in the ovaries of adult *H. axyridis*.

### DEGs analysis

Prior to differential gene expression analysis, for each sequenced library, the read counts were adjusted using the edgeR program package through one scaling normalized factor^49^. The DEGSeq R package (1.12.0) (https://bioconductor.org/packages/release/bioc/html/DESeq.html) was used to analyse differential expression under two conditions^50^. The Benjamini & Hochberg method was used to correct the P-values. Differential expression was considered significant at a P-value of 0.005 and log2 (fold change) of 1^51^ A series of genome-wide expression profiling comparisons were performed to examine gene activity changes between S1 and the other three stages. All obtained DEGs were combined into one DEG database.

### Identification and sequence analysis of development-related genes

To obtain a comprehensive perspective on the molecular basis of ovary development, we focused on the formation of the polar plasm, the signal transduction mechanism in the ovary microenvironment and yolk formation^31^. The analysis was based on the database of annotated unigenes in the ovaries of adult *H. axyridis* and the DEG database obtained in this study, literature reports^24^,^25^,^26^,^27^,^28^,^29^,^30^,^31^, and changes in gene expression levels in the four different stages to identify genes related to ovary development.

Development-related genes functional annotation clustering. Development-related genes were converted to unigenes annotation database of ovaries in H. axyridis adult and underwent functional enrichment analysis within GO and KEGG pathway terms with DAVID v6.7 (http://david.abcc.ncifcrf.gov/). The value was considered as significant when it was <0.05 for GO terms and KEGG pathways^52^.

### qRT-PCR validation of the DEGs results

A total of 20 randomly selected mRNAs were tested by qRT-PCR (Table 6). Total RNA was extracted following the RNA-seq method. Using nuclease-free water, the concentration of each RNA sample was adjusted to 1 μg/μl, and 6 μl of total RNA from each sample was used as the template to synthesize first-strand cDNA in a reverse transcription system using a first-strand synthesis kit (Reagent, Thermo Fisher Scientific, Waltham, USA) following the manufacturer’s instructions. The β-actin gene was chosen as an internal control in all qRT-PCR experiments. Twenty pairs of primers were designed to validate the RNA-seq data (Table 6). Analysis of a particular gene was performed three times under identical conditions in a 25-μl volume in the Roche Light Cycler 480 system (Reagent, Roche, Basel, Switzerland)^53^. The E (Efficiency)-method from Roche Applied Science was used to analyse the relative quantification of all target genes^53^. The expression levels of the target genes were normalized by comparison with the β-actin reference gene^53^.

## Additional Information

**How to cite this article**: Du, W. and Zeng, F. Identification of Development-Related Genes in the Ovaries of Adult *Harmonia axyridis* (Pallas) Lady Beetles Using a Time-Series Analysis by RNA-seq. *Sci. Rep.* **6**, 39109; doi: 10.1038/srep39109 (2016).

**Publisher's note:** Springer Nature remains neutral with regard to jurisdictional claims in published maps and institutional affiliations.

## Supplementary Material

Supplementary Information Tables 1–2

Supplementary Datasets 1–2

## Figures and Tables

**Table 1:** Sequencing data statistics.

Samples	Clean Reads	Clean Data	GC content (%)	% ≥Q30
Stage 1	22,364,281	5,591,070,250	41.06	93.16
Stage 2	18,460,991	4,615,247,750	40.42	92.42
Stage 3	20,636,344	5,159,086,000	39.83	92.7
Stage 4	24,531,820	6,132,955,000	37.67	92.47

Samples: the name of the sample; Clean Reads: total number of paired-end reads in the clean data; Clean Data: total number of bases in the clean data; GC content: the percentage of G and C in the total bases; % ≥Q30: the percentage of bases whose quality value was ≥30.

**Table 2:** Length range: different length ranges of the transcripts/unigenes.

Length range	Transcripts	Unigenes
300–500	28,646 (44.598%)	23,270 (48.938%)
500–1000	17,718 (27.585%)	12,919 (27.169%)
1000–2000	10,797 (16.810%)	6993 (14.707%)
2000+	7070 (11.007%)	4368 (9.186%)
Total number	64,231	47,550
Total length	61,551,345	41,919,319
N50 length	1448	1282
Mean length	958.28	881.58

**Table 3:** Unigene annotation statistics.

Annotated databases	Unigenes	≥300 bp	≥1000 bp
COG	7296	3522	3774
GO	12,985	7831	5154
KEGG	8144	4634	3510
KOG	17,235	9655	7580
Pfam	16,822	8692	8130
Swiss-Prot	16,863	9072	7791
nr	28,186	17,907	17,907
All	28,558	18,258	10,300

**Table 4:** Number of differentially expressed genes.

DEGs Set	DEGs Number	Up-regulated	Down-regulated
S1vsS2	962	134	828
S1vsS3	1334	524	810
S1vsS4	9640	4994	4646

The threshold value of significance in S1Vs S2, S1Vs S3, or S1Vs S4 were FPKM fold change >2 and FDR <0.05.

**Table 5:** Basic information for the identified development-related genes in the *H. axyridis* (Pallas) adult ovaryin nr.

Category or Unigene ID	Nucleotide length (bp)	Full length	Protein length (aa)	Homologous function in nr	Homology species & Accession number	E-value	GenBank accession numbers
**Polar formation**							
TRINITY_DN20651_c0_g14	397	Yes	102	embryonic polarity protein dorsal-like isoform X1	Polistes Canadensis L XP_015172818.1	7E-17	KY020084
**Signal transduction mechanism**							
**Bone morphogenetic protein function related**							
TRINITY_DN19519_c1_g1	3186	Yes	517	bone morphogenetic protein receptor type-1B isoform X1	*Agrilus planipennis* Fairmaire XP_018576589.1	0.0	KY020085
TRINITY_DN20146_c0_g1	4758	Yes	821	bone morphogenetic protein receptor type-2	*Tribolium castaneum* Herbst XP_974821.1	0.0	KY020086
**DE-cadherin function related**							
TRINITY_DN17181_c3_g1	4956	Yes	1444	DE cadherin protein	*Anoplophora glabripennis* Motschulsky XP_018578727.1	0.0	KY020087
**Insulin function related**							
TRINITY_DN19612_c0_g1	2222	5′	737	insulin receptor substrate 1-like	*Agrilus planipennis* Fairmaire XP_018322097.1	0.0	KY020088
TRINITY_DN19612_c0_g9	352	5′	105	insulin receptor substrate 1 isoform X6	*Tribolium castaneum* Herbst XP_008196601.1	3E-13	KY020089
**Ecdysone signalling pathways**							
TRINITY_DN20112_c0_g2	3802	Yes	792	ecdysone-induced protein 75B, isoforms C/D isoform X3	*Anoplophora glabripennis* Motschulsky XP_018561963.1	0.0	KY020090
**Notch signalling pathway**							
TRINITY_DN19749_c1_g1	2905	Yes	379	fringe glycosyltransferase	*Tribolium castaneum* Herbst XP_008198283.1	4E-173	KY020091
TRINITY_DN20315_c0_g1	3282	Yes	792	CREB-binding protein isoform X3	*Tribolium castaneum* Herbst XP_008192362.1	0.0	KY032003
TRINITY_DN20898_c0_g2	453	3′	142	histone acetyltransferase KAT2A	*Tribolium castaneum* Herbst XP_969631.2	7E-49	KY020092
TRINITY_DN20651_c0_g18	400	3′	119	Dorsal1	*Tribolium castaneum* Herbst EFA02850.1	8E-48	KY031985
TRINITY_DN20799_c0_g3	3812	5′	1135	tight junction protein ZO-2 isoform X3	*Anoplophora glabripennis* Motschulsky XP_018564773.1	0.0	KY031986
TRINITY_DN18711_c0_g1	1845	5′	543	neurogenic locus Notch protein	*Anoplophora glabripennis Motschulsky* XP_018574509.1	0.0	KY031987
TRINITY_DN9215_c0_g1	1518	Yes	100	gamma-secretase subunit pen-2	*Tribolium castaneum.* Herbst XP_008194720.1	2E-42	KY031988
TRINITY_DN15150_c0_g1	3585	Yes	884	protein strawberry notch isoform X1	*Tribolium castaneum* Herbst XP_015836321.1	0.0	KY031989
TRINITY_DN21369_c1_g1	6522	Yes	375	notch isoform X2	*Tribolium castaneum* Herbst XP_008200304.1	0.0	KY031990
TRINITY_DN17863_c0_g1	4186	Yes	1285	protein jagged-1b isoform X2	*Tribolium castaneum* Herbst XP_008196297.1	0.0	KY031991
TRINITY_DN20651_c0_g5	561	5′	185	Dorsal2	*Tribolium castaneum* Herbst NP_001034507.1	8E-57	KY031993
**Jak-STAT signalling pathway**							
TRINITY_DN15528_c0_g1	1444	Yes	279	suppressor of cytokine signalling 2	Tribolium castaneum. Herbst XP_008196669.1	1E-79	KY031994
TRINITY_DN17727_c1_g1	1766	Yes	481	RNA-binding protein 41	Tribolium castaneum. Herbst XP_008191526.1	5E-39	KY031995
TRINITY_DN18156_c0_g1	3760	Yes	380	sprouty-related protein with EVH-1 domain isoform X3	*Tribolium castaneum.*Herbst XP_015833056.1	6E-145	KY031996
TRINITY_DN20474_c0_g2	2362	Yes	250	protein sprouty	*Tribolium castaneum*Herbst XP_008192086.1	3E-70	KY031997
TRINITY_DN20711_c0_g4	2083	5′	589	phosphatidylinositol 4,5-bisphosphate 3-kinase catalytic subunit delta isoform	*Agrilus planipennis* Fairmaire XP_018318828.1	2E-50	KY031999
**Vitellogenin function related**							
TRINITY_DN20508_c0_g4	308	Yes	34	vitellogenin receptor	*Tribolium castaneum*Herbst XP_015837722.1	3E-35	KY032000
TRINITY_DN20502_c0_g1	5471	Yes	1785	vitellogenin	*Tribolium castaneum*Herbst XP_971398.1	0.0	KY032001
TRINITY_DN20736_c0_g1	5505	Yes	1791	vitellogenin	*Tribolium castaneum* Herbst XP_971398.1	0.0	KY032002

**Table 6:** Sequences of the primers for qRT-PCR analysis of 20 random genes.

GeneID	Location of primer	Primer	Product size	Tm	Expression Level
β-actin	F	CTATGTCGGAGCCCATCACT	112	60	
	D	AGCAGTTGTAGCTTCTCCGT			
TRINITY_DN20799_c0_g3	F	GATCCCAGTGTTGTGATGGC	113	60	down
	D	GCGTGTCTGGTTCTGCTATG			
TRINITY_DN20651_c0_g5	F	TTATATAACACGCTTCTAAG	124	60	up
	D	TCCTGAGGAACTTGTTGTTC			
TRINITY_DN21369_c1_g1	F	CAACAACAACGGTACCTGCA	105	60	down
	D	CGAGCAAGGGTTGGATAAGC			
TRINITY_DN17863_c0_g1	F	TGGTGGGCGATTATGTCTGT	126	60	down
	D	GGCAAGCACAGTGATAGTCG			
TRINITY_DN20651_c0_g13	F	CCTCACCCTCACAACTTGGT	105	60	up
	D	TACATCCCTCACGACCAACC			
TRINITY_DN17712_c0_g1	F	AGAGAACCAACCCCATCTGG	118	60	up
	D	TCTCATCACCTTCGAGCTCC			
TRINITY_DN20851_c0_g4	F	AGCCTTTGTACTCGGTTCTGA	111	60	down
	D	GCCAGTGAAATCCGGTCTTC			
TRINITY_DN19101_c0_g2	F	GGATCAGCAAATATTTCTGGGGA	118	60	up
	D	ACCAGAGGTCCCCAGAAATA			
TRINITY_DN19788_c0_g2	F	TAGAGAAGCCAGGCGACAAA	124	60	up
	D	CGGCACCCATTAACAGGATC			
TRINITY_DN18367_c0_g4	F	CCATCGTAGCGCCATTTACC	98	60	down
	D	ATTGGGGTAGTTGGCGAAGT			
TRINITY_DN13604_c0_g1	F	GACCAGGGCTTGTTCCAAAG	124	60	down
	D	CTCTCTGCTGAACCTTTCGC			
TRINITY_DN19101_c0_g1	F	CTGGAATGGTTGGTGGGTTC	117	60	up
	D	AACGCTGCCATGTTTCCAAT			
TRINITY_DN16334_c3_g1	F	TCTCGTCCCAGTACGATTCG	113	60	down
	D	TGACTTTGGCACGTTCACAG			
TRINITY_DN6859_c0_g1	F	GATCTCCGTTTCCAATCGGC	114	60	up
	D	GACACGCTTGGCATGGATAG			
TRINITY_DN21302_c0_g2	F	TGAGGAATGGGAGGAGGTCT	145	60	down
	D	CCATTCCTCATCCACCCGG			
TRINITY_DN13782_c0_g1	F	TGGTCCGTACAAAGCAAACG	136	60	up
	D	ACGGAATCTGTGGGGTCTTT			
TRINITY_DN27602_c0_g1	F	ACTTCACACGGATTTGCGAG	120	60	up
	D	GTTTGCTAAGATCGCCAGGG			
TRINITY_DN19725_c0_g1	F	GGTGGAAGTGAAGCGCAAAA	96	60	down
	D	GTCTCCCAAACTCTCCTCGA			
TRINITY_DN11810_c0_g1	F	GAGCCAAGCACTTCGAGATG	115	60	up
	D	TCCACTGATCAAACTGGCGA			
TRINITY_DN15723_c0_g2	F	TGGTGTCTGATTGTTTGGCG	134	60	up
	D	TCGTCAGATATGGGTACGTCC			

F indicates forward primer, and D indicates reverse primer. Tm indicates the temperature at which 50% of the primers and complementary sequence form the double-stranded DNA molecule.
