# Supplementary material for: Identification of Development-Related Genes in the Ovaries of Adult Harmonia axyridis (Pallas) Lady Beetles Using a Time- Series Analysis by RNA-seq
Source: Sci Rep. 2016 Dec 14;6:39109. doi: 10.1038/srep39109 (PMC5155419; doi:10.1038/srep39109)
Supplement: Supplementary Information Tables 1–2 [file srep39109-s1.pdf]

Identification of Development-Related Genes in the Ovaries of Adult *Harmonia axyridis* (Pallas) Lady Beetles Using a Time- Series Analysis by RNA-seq

WenxiaoDu[1], Fanrong Zeng[1*]

Supplementary information Table 1. GO annotation classification of development-related genes converted to unigenes annotation database of ovaries in *H. axyridis* adult

GO ID	Description	GeneRatio	BgRatio	Pvalue	Fdr
Biological Process					
GO:0007219	Notch signaling pathway	6(35.29%)	24(0.25%)	1.55E-12	8.69E-10
GO:0007166	cell surface receptor signaling pathway	7(41.18%)	176(1.84%)	1.06E-08	2.98E-06
GO:0050794	regulation of cellular process	14(82.35%)	2090(21.87%)	1.91E-07	3.57E-05
GO:0050789	regulation of biological process	14(82.35%)	2273(23.79%)	5.79E-07	8.12E-05
GO:0065007	biological regulation	14(82.35%)	2389(25%)	1.12E-06	0.000125109
GO:0007165	signal transduction	9(52.94%)	901(9.43%)	6.85E-06	0.000640691
GO:0023052	signaling	9(52.94%)	1021(10.68%)	1.92E-05	0.001347786
GO:0044700	single organism signaling	9(52.94%)	1021(10.68%)	1.92E-05	0.001347786
GO:0007154	cell communication	9(52.94%)	1044(10.93%)	2.31E-05	0.001437517
GO:0051716	cellular response to stimulus	9(52.94%)	1066(11.16%)	2.73E-05	0.00153374
GO:0050896	response to stimulus	10(58.82%)	1529(16%)	7.02E-05	0.003581689
GO:0007275	multicellular organism development	8(47.06%)	932(9.75%)	8.65E-05	0.004045346
GO:0044707	single-multicellular organism process	8(47.06%)	988(10.34%)	0.00013138	0.005669553
GO:0044767	single-organism developmental process	8(47.06%)	1106(11.57%)	0.00029166	0.01095999
GO:0032501	multicellular organismal process	8(47.06%)	1115(47.67%)	0.00030869	0.01095999
GO:0032502	developmental process	8(47.06%)	1117(11.69%)	0.000312584	0.01095999
GO:0006355	regulation of transcription, DNA-templated	6(35.29%)	607(6.35%)	0.000433129	0.012788717
GO:1903506	regulation of nucleic acid-templated transcription	6(35.29%)	607(6.35%)	0.000433129	0.012788717
GO:2001141	regulation of RNA biosynthetic process	6(35.29%)	607(6.35%)	0.000433129	0.012788717
GO:0051252	regulation of RNA metabolic process	6(35.29%)	623(6.52%)	0.000498342	0.0139785
GO:0019219	regulation of nucleobase-containing compound metabolic process	6(35.29%)	637(6.67%)	0.000561556	0.015001555
GO:0010556	regulation of macromolecule biosynthetic process	6(35.29%)	662(6.93%)	0.000690038	0.016830925
GO:2000112	regulation of cellular macromolecule biosynthetic process	6(35.29%)	662(6.93%)	0.000690038	0.016830925
GO:0009889	regulation of biosynthetic process	6(35.29%)	676(7.07%)	0.00077147	0.017311776
GO:0031326	regulation of cellular biosynthetic process	6(35.29%)	676(7.07%)	0.00077147	0.017311776
GO:0051171	regulation of nitrogen compound metabolic	6(35.29%)	697(7.29%)	0.000907567	0.019582507

	process				
GO:0010468	regulation of gene expression	6(35.29%)	706(7.39%)	0.000971352	0.020182534
GO:0023014	signal transduction by protein phosphorylation	2(11.76%)	28(0.29%)	0.001095814	0.021955411
GO:0050793	regulation of developmental process	4(23.53%)	278(2.91%)	0.00123642	0.023626208
GO:0006473	protein acetylation	2(11.76%)	32(0.33%)	0.001431891	0.023626208
GO:0006475	internal protein amino acid acetylation	2(11.76%)	32(0.33%)	0.001431891	0.023626208
GO:0016573	histone acetylation	2(11.76%)	32(0.33%)	0.001431891	0.023626208
GO:0018393	internal peptidyl-lysine acetylation	2(11.76%)	32(0.33%)	0.001431891	0.023626208
GO:0018394	peptidyl-lysine acetylation	2(11.76%)	32(0.33%)	0.001431891	0.023626208
GO:0043543	protein acylation	2(11.76%)	35(0.37%)	0.001712313	0.025590044
GO:0000059	protein import into nucleus, docking	1(5.88%)	1(0.01%)	0.001778987	0.025590044
GO:0006607	NLS-bearing protein import into nucleus	1(5.88%)	1(0.01%)	0.001778987	0.025590044
GO:0072697	protein localization to cell cortex	1(5.88%)	1(0.01%)	0.001778987	0.025590044
GO:1990778	protein localization to cell periphery	1(5.88%)	1(0.01%)	0.001778987	0.025590044
GO:0001708	cell fate specification	2(11.76%)	39(0.41%)	0.00212358	0.029783207
GO:0080090	regulation of primary metabolic process	6(35.29%)	842(8.81%)	0.002434228	0.032855302
GO:0007293	germarium-derived egg chamber formation	2(11.76%)	42(0.44%)	0.002459755	0.032855302
GO:0060255	regulation of macromolecule metabolic process	6(35.29%)	852(8.92%)	0.00258624	0.033741407
GO:0031323	regulation of cellular metabolic process	6(35.29%)	863(9.03%)	0.002761765	0.035212498
GO:0036059	nephrocyte diaphragm assembly	1(5.88%)	2(0.02%)	0.003554995	0.044318939
Molecular Function					
GO:0004675	transmembrane receptor protein serine/threonine kinase activity	2(12.5%)	6(0.05%)	2.92E-05	0.002894087
GO:0005509	calcium ion binding	5(31.25%)	341(3.08%)	8.86E-05	0.004223252
GO:0005112	Notch binding	2(12.5%)	12(0.11%)	0.000127977	0.004223252
GO:0019199	transmembrane receptor protein kinase activity	2(12.5%)	14(0.13%)	0.000176156	0.004359873
GO:0001071	nucleic acid binding transcription factor activity	4(25%)	250(2.26%)	0.000371971	0.006137528
GO:0003700	transcription factor activity, sequence-specific DNA binding	4(25%)	250(2.26%)	0.000371971	0.006137528
GO:0004402	histone acetyltransferase activity	2(12.5%)	29(0.26%)	0.000776061	0.009603757
GO:0061733	peptide-lysine-N-acetyltransferase activity	2(12.5%)	29(0.26%)	0.000776061	0.009603757
GO:0034212	peptide N-acetyltransferase activity	2(12.5%)	32(0.29%)	0.000945702	0.010402727
GO:0033829	O-fucosylpeptide 3-beta-N-acetylglucosaminyltransferase activity	1(6.25%)	1(0.01%)	0.001444174	0.014297319
GO:0008080	N-acetyltransferase activity	2(12.5%)	46(0.42%)	0.001950272	0.017552449
GO:0016407	acetyltransferase activity	2(12.5%)	50(0.45%)	0.002300537	0.018979428
GO:0016410	N-acyltransferase activity	2(12.5%)	58(0.52%)	0.003083494	0.023481993
GO:0046872	metal ion binding	8(50%)	1975(17.83%)	0.003321355	0.023486722
GO:0043169	cation binding	8(50%)	1996(18.02%)	0.003558858	0.023488463
GO:0038023	signaling receptor activity	3(18.75%)	256(2.31%)	0.005464012	0.027177724
GO:0008320	protein transmembrane transporter activity	1(6.25%)	4(0.04%)	0.005764972	0.027177724
GO:0019955	cytokine binding	1(6.25%)	4(0.04%)	0.005764972	0.027177724

GO:0022884	macromolecule transmembrane transporter activity	1(6.25%)	4(0.04%)	0.005764972	0.027177724
GO:0050699	WW domain binding	1(6.25%)	4(0.04%)	0.005764972	0.027177724
GO:0070379	high mobility group box 1 binding	1(6.25%)	4(0.04%)	0.005764972	0.027177724
GO:0005024	transforming growth factor beta-activated receptor activity	1(6.25%)	5(0.05%)	0.007201338	0.032406023
GO:0008375	acetylglucosaminyltransferase activity	1(6.25%)	7(0.06%)	0.010068237	0.043337195
Cellular Component					
GO:0016020	membrane	13(81.25%)	2457(42.48%)	0.001812643	0.107363878
GO:0005917	nephrocyte diaphragm	1(6.25%)	2(0.03%)	0.005525328	0.107363878
GO:0036056	filtration diaphragm	1(6.25%)	2(0.03%)	0.005525328	0.107363878
GO:0005912	adherens junction	2(12.5%)	45(0.78%)	0.0066274	0.107363878
GO:0070161	anchoring junction	2(12.5%)	45(0.78%)	0.0066274	0.107363878
GO:0005768	endosome	2(12.5%)	53(0.92%)	0.009106767	0.117199631
GO:0005911	cell-cell junction	2(12.5%)	56(0.97%)	0.010128363	0.117199631
GO:0070765	gamma-secretase complex	1(6.25%)	6(0.1%)	0.01649023	0.166963582
GO:0044425	membrane part	9(56.25%)	1683(29.1%)	0.020868816	0.177633651
GO:0005913	cell-cell adherens junction	1(6.25%)	8(0.14%)	0.02193008	0.177633651
GO:0055037	recycling endosome	1(6.25%)	12(0.21%)	0.032725265	0.219472754
GO:0030139	endocytic vesicle	1(6.25%)	15(0.26%)	0.040748239	0.219472754
GO:0005634	nucleus	7(43.75%)	1271(21.97%)	0.042686369	0.219472754
GO:0044459	plasma membrane part	3 (18.75%)	291(5.03%)	0.043377148	0.219472754
GO:0005643	nuclear pore	1 (6.25%)	16(0.28%)	0.04340867	0.219472754
GO:0035003	subapical complex	1(6.25%)	16(0.28%)	0.04340867	0.219472754
GO:0005770	late endosome	1(6.25%)	17(0.29%)	0.046062183	0.219472754
GO:0030054	cell junction	13(81.25%)	2457(42.48%)	0.054512175	0.245304786

Supplementary information Table 2. KEGG pathway annotation of development-related genes converted to unigenes annotation database of ovaries in *H. axyridis* adult

Pathway	DEGs genes with pathway annotation	All genes with pathway annotation	Pvalue	Qvalue	Pathway ID
Notch signaling pathway	7 (53.85%)	62 (1.29%)	0.000000	0.000000	ko043302
FoxO signaling pathway	5 (38.46%)	125 (2.6%)	0.000012	0.000077	ko040683
mTOR signaling pathway	4 (30.77%)	75 (1.56%)	0.000035	0.000151	ko041504
Longevity regulating pathway - multiple species	4 (30.77%)	94 (1.95%)	0.000085	0.000276	ko042135
Insulin resistance	4 (30.77%)	104 (2.16%)	0.000126	0.000328	ko049316
Jak-STAT signaling pathway	3 (23.08%)	43 (0.89%)	0.000178	0.000386	ko046307
TGF-beta signaling pathway	3 (23.08%)	79 (1.64%)	0.001079	0.002005	ko043508
Dorso-ventral axis formation	2 (15.38%)	56 (1.16%)	0.009542	0.015506	ko043209
AGE-RAGE signaling pathway in diabetic complications	2 (15.38%)	60 (1.25%)	0.010901	0.015746	ko0493310
